# Newborn Screening Samples for Diabetes Research: An Underused Resource

**DOI:** 10.3390/cells9102299

**Published:** 2020-10-15

**Authors:** Jane Frances Grace Lustre Estrella, Jincy Immanuel, Veronica Wiley, David Simmons

**Affiliations:** 1 School of Medicine, Macarthur Clinical School, Western Sydney University, Sydney, NSW 2560, Australia; J.Estrella2@westernsydney.edu.au (J.F.G.L.E.); J.Varghese@westernsydney.edu.au (J.I.); 2 NSW Newborn Screening Program, Sydney, NSW 2145, Australia; veronica.wiley@health.nsw.gov.au; 3 Faculty of Medicine and Health, The University of Sydney Children’s Hospital Westmead Clinical School, Sydney, NSW 2145, Australia

**Keywords:** newborn screening, diabetes mellitus, dried blood spots, amino acids, metabolites, type 1 diabetes

## Abstract

Inborn errors of metabolism and diabetes share common derangements in analytes of metabolic networks that are tested for in newborn screening, usually performed 48–72 h after birth. There is limited research examining the metabolic imprint of diabetes on newborn screening results. This paper aims to demonstrate the links between diabetes, biochemical genetics and newborn screening in investigating disease pathophysiology in diabetes, provide possible reasons for the lack of research in diabetes in newborn screening and offer recommendations on potential research areas. We performed a systematic search of the available literature from 1 April 1998 to 31 December 2018 involving newborn screening and diabetes using OVID, MEDLINE, Cochrane and the PROSPERO register, utilizing a modified extraction tool adapted from Cochrane. Eight studies were included after screening 1312 records. Five studies reanalyzed dried blood spots (DBS) on filter paper cards, and three studies utilized pre-existing results. The results of these studies and how they relate to cord blood studies, the use of cord blood versus newborn screening dried blood spots as a sample and considerations on newborn screening and diabetes research is further discussed. The timing of sampling of newborn screening allows insight into neonatal physiology in a catabolic state with minimal maternal and placental influence. This, combined with the wide coverage of newborn screening worldwide, may aid in our understanding of the origins of diabetes.

## 1. Introduction

Despite their rarity, inborn errors of metabolism (IEM) share commonalities with diabetes, such as insulin resistance, hyperglycemia and acidosis and target-organ damage [1,2]. Both IEM and diabetes involve derangements in the intermediates of protein, carbohydrate and lipid metabolic pathways. Analytes routinely tested for in newborn screening (NBS) for IEM, 48–72 h after birth, include many of the metabolites involved in these metabolic pathways. Some of the analytes tested for in NBS are deranged in diabetes overall [3,4,5,6,7] and diabetes in pregnancy [8,9,10,11,12,13,14,15]; however, these studies utilized cord blood (Table 1). Cord blood as a sample for neonatal metabolic derangements has been shown to give false-negative results for amino acid and lipid disorders and for diagnosing maternal IEM in leucine, carnitine and vitamin-dependent pathways [16,17,18], reflecting placental/maternal metabolism for some analytes. Cord blood may, therefore, not give a complete picture of neonatal metabolic networks.

Residual deidentified dried blood spots (DBS) derived from routine NBS form a biobank and have been used in a few diabetes studies (mostly from Europe) and have given insight into the disease pathophysiology. Recent exciting publications from the Hyperglycemia and Adverse Pregnancy Outcomes study (HAPO), using cord blood [11,15], have highlighted the potential importance of intermediate metabolites in the pathophysiology of diabetes. The question arises how much of this pattern is neonatal and whether similar analyses using NBS could provide additional insights. This article will summarize the available literature on NBS and diabetes, illustrate how the results of NBS may complement cord blood-based research, provide insight on reasons behind the lack of secondary research using analyzed NBS results and make recommendations regarding future research.

## 2. A Brief History of Newborn Screening

This widely successful public health initiative began as a diagnostic test for intellectual disability. In 1934, Ivan Asbjørn Følling, a Norwegian physician, demonstrated the presence of phenolpyruvic acid (a ketoacid intermediate of phenylalanine metabolism) in the urine of a child with intellectual disability who was normal at birth. The urine, which had a strong, musty odor, turned greenish-blue following the addition of ferric chloride [19]. This formed the basis of the “nappy test”, performed on infants at six weeks to diagnose phenylketonuria in the 1950s. Robert Guthrie’s bacterial inhibition assay involving special filter paper revolutionized the field, allowing earlier pre-symptomatic diagnosis and treatment. The introduction of mass spectrometry in the late 1990s/early 2000 allowed the simultaneous screening of multiple target disorders from the same sample. Today, NBS’s success stems from saving infant lives by averting metabolic crises, preventing intellectual disability through pre-symptomatic treatment in affected infants and overall health economic benefits [20].

The primary purpose of NBS is screening for serious conditions that present with reasonable frequency and require treatment early in life. Coverage approaches >99.9% in most developed countries, with the test offered to all newborns [18]. While most countries worldwide have NBS programs, screened target disorders, procedure and policy vary across locations, with some countries screening for one disorder while others screen for up to 28. The UK screens for nine target disorders on day five of life, while most countries screen at 48–72 h [21,22]. Six of these disorders (phenylketonuria, medium-chain acyl-CoA-dehydrogenase deficiency, maple syrup urine disease, isovaleric acidemia, glutaric aciduria type 1 and pyridoxine-unresponsive homocystinuria) utilize mass spectrometry techniques and have biochemical intermediates associated with diabetes (Table 2). In contrast, Australia screens at 48–72 h of life for over 28 target disorders using tandem mass spectrometry (MS/MS).

## 3. Available Literature on Diabetes and Newborn Screening

The use of NBS in diabetes research to date may be summarized as follows: risk prediction for type 1 diabetes development, determining metabolic perturbations in infants exposed to maternal diabetes, confirming the metabolite signature of a recently discovered monogenic diabetes gene and determining the feasibility of testing neonatal diabetes [23,24,25,26,27,28,29,30]. These studies utilized either pre-existing results, reused and reanalyzed DBS (using the card as a biobank) for new biomarkers or a combination of both. 

Several studies have looked at the metabolic signature of diabetes in infants born to mothers with diabetes in pregnancy [8,9,10,11,12,13,14,15]. However, these studies utilized cord blood collected at birth, which may not reflect neonatal metabolism that is independent of maternal/placental influences for some analytes. We sought to determine the studies that have used NBS to examine the effects of diabetes on the neonatal milieu. The detailed methodology, search terms used and summary of the search are available in the Supplement Section. We hand-searched 1312 titles and abstracts and chose eight studies for inclusion (Table 3). All studies were conducted in Europe. One article from the United States found during a grey search was excluded, as there was no full-text report available. A study testing for antibodies associated with type 1 diabetes development using punch-outs from DBS of neonates [31] was excluded, as these markers were not metabolites per se but antibodies that were well-known risk determinants for type 1 diabetes.

Five studies reused and reanalyzed dried blood spots, and three studies utilized pre-existing results. Of the five studies that reanalyzed the DBS, one reported an association between type 1 diabetes development and infant iron levels. A two-fold increase of developing type 1 diabetes for each doubling of iron content was found (cases: mean iron content 1.80 (SD 0.30) units, controls: 1.74 (SD 0.39); odds ratio (OR) 2.07 (95% CI; 1.07; 4.00) (*p* ≤ 0.030), which strengthened after adjusting for confounders (OR 2.55; 1.04; 6.24 95% CI) (*p* ≤ 0.041). These results had public health implications, as there was a trend towards increased iron contents on DBS in later years (1993–1998), thought to be due to increased supplementation during pregnancy [23]. The second study reported a reanalysis of dried blood spots to test glucose levels for neonatal diabetes and established median capillary glucose levels for neonatal diabetes (cases vs. matched controls: 18.7 (10.2 to >30.0) vs. 4.6 (2.3–6.2) mmol/L, *p* ≤ 0.001), demonstrating that newborn screening for neonatal diabetes was feasible [1,24]. Two studies showed no associations between vitamin D [25,26] or zinc concentrations [27] and type 1 diabetes development. 

Three of the eight studies examined pre-existing results and did not use the DBS as a biobank. One retrospective case control study utilizing pre-existing newborn screening results showed an association between low acylcarnitine levels and type 1 diabetes [2,28]. A study from Spain demonstrated a trend of higher total carnitine, free carnitine and short and medium-chain acylcarnitines in infants exposed to gestational diabetes (GDM), which paralleled some of the results of the HAPO cohort on cord blood [11] but found no differences in acylcarnitines between mothers who were on dietary treatments versus those on insulin [29]. Finally, molecular work to identify new genes involved in diabetes showed hyperphenylalaninemia on newborn screening in patients with *PCBD1* mutations, demonstrating how multiple metabolic networks can influence diabetes development [30].

In summary, the available literature on diabetes research using routine NBS results has shown that (1) future type 1 diabetes patients have lower acylcarnitines when compared to controls, (2) gestational diabetes exerts some influence on infant metabolism manifesting as higher carnitine and acylcarnitine fractions on NBS as compared to unexposed infants and (3) higher phenylalanine levels on routine NBS results are seen in patients with *PCBD1* mutations (some of whom were diagnosed with type 2 diabetes later in life). These analytes, routinely assessed for in NBS, may serve as biomarkers for both diabetes and inborn errors of metabolism. The use of NBS DBS for a secondary analysis has involved the analysis of iron, zinc and vitamin D for future type 1 diabetes risk and the feasibility and stability of a glucose analysis for detecting neonatal diabetes.

## 4. Studies Using Cord Blood in Diabetes Research Evaluating Metabolite Derangements 

We focused our literature search specifically on cord blood studies that (1) evaluated the metabolic imprint at birth of patients who developed type 1 diabetes and (2) analyzed trends and metabolic perturbations in infants born to mothers with diabetes in pregnancy (Table 1). Surprisingly, there were no studies that determined the effects of pharmacologic diabetes treatment in cord blood acylcarnitines and amino acids. 

Lower levels of phospholipids (phosphatidylcholine) were a consistent finding in studies of cord blood of infants who developed type 1 diabetes [3,4,5,6,7]. The metabolic perturbations in the cord blood of infants born to women with GDM varied among ethnicities, with derangement in BCAA (Branched Chain Amino Acids) and intermediates the most common affected networks [8,9,10,11,12,13,14,15].

## 5. Newborn Screening Dried Blood Spots vs. Cord Blood in Diabetes Research: Advantages and Disadvantages as a Testing Sample 

### 5.1. Sample Volume and Storage

The small amounts of blood volume needed in newborn screening (50–75 μL per blood spot) provides an additional advantage for population-based screening: once dry, the blotted filter paper can be sent via mail from collection centers to the testing laboratory, avoiding the need for couriers. The attractiveness of using DBS has filtered from NBS to mainstream medicine, finding uses such as HbA1c monitoring, maternal research, infection diagnosis, drug monitoring, a source for DNA postmortem (identification and diagnosis) and the like [32,33,34,35,36,37]. 

Sample volumes of DBS limit its use in untargeted metabolomic techniques. Furthermore, variables such as patient hematocrit, temperature, humidity and storage conditions contribute to measurements of uncertainty [17]. Importantly, McDonald and colleagues have demonstrated the feasibility of a retrospective diagnosis of neonatal diabetes using stored NBS cards [24] and that elevated glucose levels may be reliably detected in various storage conditions at day five of life.

Cord blood has the advantage of greater sample volume (between 60–110 mL), allowing its use in both targeted and untargeted metabolomic techniques and include identify analytes not typically tested in NBS. Components of cord blood such as plasma may be used for analysis, without the need to test or correct for hematocrit. A controlled set temperature of between −70 to −20 °C is necessary, but the rigid storage conditions dictated minimize measurements of uncertainty. It is possible to blot cord blood into filter paper for analysis, but the sample becomes subject to the same conditions as the DBS in NBS with similar measurements of uncertainty. 

Other practical issues can also impact on the availability of collection. For example, cord blood has other clinical uses (e.g., banking for stem cell transplant), and volumes can be limited under some circumstances, while a neonatal heel prick is generally only used for IEM screening and is universally collected. NBS is now part of clinical care, so it adds no additional work burden on clinical staff, while cord blood collection remains a nonroutine endeavor unless an explicit local policy (e.g., for research purposes). 

### 5.2. Time of Sampling

Multiple studies in NBS have demonstrated compromised sensitivity and specificity for detecting target disorders if samples are collected immediately after birth [16,17,18]. This is due to the physiologic variability of normal ranges in analytes occurring immediately after birth, during the transition from placental/maternal metabolism to neonatal metabolism. For example, thyrotropin (thyroid stimulating hormone; TSH) demonstrates a physiologic surge after birth, and results of early sampling may give a false-positive result for congenital hypothyroidism. Phenylalanine and tyrosine, reported as deranged in some studies of cord blood and GDM, also vary widely from as early as the first hour of life [9,11]. It is due to the hourly and daily variability of some of these analytes that DBS are collected typically at 48–72 h after birth, facilitating better pick-up rates for disorders of fatty oxidation. The practice in Australia is to request resampling if samples are collected too soon after birth.

Umbilical cord sampling usually takes place at birth involving the immediate collection of larger blood volumes. Paired samples of umbilical cord and maternal blood may provide information on differences between the maternal and fetal metabolome [11,15] and demonstrate the physiologic differences that surround maternal and fetoplacental metabolism. The drawback of using cord blood (or any blood sample) drawn at birth is that it may not provide accurate information on neonatal physiology that is independent of maternal influence [16,17,18]. 

These points have been summarized in Table 4.

### 5.3. Analytes Assessed

Testing for multiple disorders of carbohydrate, amino acid and fat metabolism is undertaken simultaneously on the same DBS in NBS. A list of target disorders and their analytes and selected studies utilizing metabolomics that have tested for similar analytes on cord blood may be found in Table 2. It is important to note that whole blood is analyzed in the DBS sample, while cord blood, depending on which method is utilized, may use whole blood or plasma. This distinction is important: not all analytes are equimolar between plasma and its cellular components. For example, carnitine and its esters are higher in concentration in red blood cells than in plasma [38]. Cord blood samples have the advantage of being able to test known (targeted) and unknown (untargeted) analytes using either whole blood or plasma. Modeling for metabolic networks can also then be undertaken.

## 6. Examples of How Cord Blood Studies in Diabetes and Newborn Screening Studies May Complement Each Other

Outcomes of studies that have utilized newborn screening results may not be comparable with studies that have used cord blood due to differences such as sample type (whole blood versus plasma), sample size, volume, timing of sample collection and the like. However, it is possible to demonstrate how the results of one study may complement the other. An example are the studies from Finland and Sweden exploring phospholipid levels in cord blood [3,4,5,6,7]. A consistent finding was decreased phosphatidylcholine levels in the cord blood of selected at-risk Finnish and Swedish children who developed type 1 diabetes before the age of 10 years independent of the genetic risk and β-cell autoimmunity. Phosphatidylcholine is an essential component of the phospholipid membrane of cells and the mitochondrial membrane, while the carnitine-transport shuttle allows long-chain fatty acids to enter these membranes for energy generation in the mitochondrial cytosol. It is interesting to note that a separate study using NBS results demonstrated low total and free carnitine levels in children who developed type 1 diabetes before six years [28]. This provides more evidence that a child’s immune system is shaped by many factors, some of which may be early in life. Conversely, we do not know if carnitine has a direct role in the pathogenesis of β-cell loss. Replicating this study may provide some answers on the rising epidemiology [39] of type 1 diabetes.

The Hyperglycemia and Adverse Pregnancy Outcomes (HAPO) was a large, multi-center trial that established continuous associations between birth weight and maternal glucose levels below the threshold for diabetes. There have been two studies using cord blood metabolomics from this cohort: one looked at associations between maternal body mass index (BMI) and glucose levels and cord blood metabolites and associations between cord blood metabolites and newborn birth weight and adiposity [11], and another study looked at cord blood metabolites, metabolite networks and newborn adiposity and insulin resistance [15]. 

The first study used a targeted metabolomic technique and looked at 1600 maternofetal dyads of different ancestries, establishing associations between maternal phenotypes and cord blood metabolites and cord blood metabolites and newborn outcomes. 

The second study used both targeted and untargeted metabolomic techniques with modeling in investigating metabolic networks in cord blood and newborn outcomes, specifically the birth weight and sum of skinfolds (SSF), a marker of fetal adiposity.

The results of both studies indicated that, in addition to associations drawn between maternal phenotypes, cord metabolites (some of which are tested for in NBS, such as various acylcarnitines and branched-chain amino acids) and newborn outcomes, a distinctive metabolic phenotype unique to each ancestral group was found. There was a positive correlation between fasting plasma glucose (FPG) and cord blood butyrylcarnitine (carnitine ester of 3-hydroxybutyrate; AC C4OH) and cord blood C-peptide and medium and long-chain acylcarnitines in Mexican American infants. North European ancestry was associated with a positive correlation between cord blood SSF and 3-hydroxybutyrate after adjusting for covariates that included the maternal phenotype (BMI, FPG, age, etc.); newborn outcomes (gestational age at delivery, sex, etc.) and cord blood c-peptide and a negative association with cord c-peptide and medium and long-chain acylcarnitines, the palmitoleate/palmitate ratio and palmitoleic acid. A negative association was seen in Thai ancestry between long-chain acylcarnitine AC C20-OH/C18-DC and BW and AC C20-OH/C18-DC, triglycerides and SSF after adjustments for C-peptide. C-peptide and C16, the palmitoleic/palmitate ratio and oleic acid/steric acid ratio were also negatively associated with Thai ancestry. African-Caribbean ancestry showed positive associations with certain cord blood medium-chain acylcarnitines (AC C8:1, AC C10:3, AC C10:1 and AC C10:2); histidine; glycine; 3-hydroxybutyrate and AC C4OH; palmitoleic/palmitate ratio with BW and SSF (after covariate adjustment). A negative association was found between c-peptide and 1, 5 anhydroglucitol, 3-hydroxybutyrate, certain medium and long-chain acylcarnitines, the palmitoleic/palmitate ratio, oleic/stearic acid and, after covariate adjustment, a negative correlation with AC C4/Ci4 and SSF. 

It would be useful to see if some of these analytes (for example, leucine/isoleucine and acylcarnitine species) that are tested on DBS persist beyond birth to day three during NBS sampling and if similar associations persist. The neonatal period is a time of intense catabolic activity, where the infant is exposed to short periods of fasting (as opposed to the intrauterine environment, where there is a constant substrate supply from the placenta), and any detectable differences, if present, may provide further information that would deepen our understanding of intrauterine glycemic exposure. Kadakia and colleagues theorized the accumulation of substrate leading to a mitochondrial metabolic gridlock, where ineffective fuel switching leads to tissue and plasma accumulation of fatty acids and influencing insulin sensitivity. It is important to remember that peroxisomes are primarily involved in fatty acid oxidation and lipid biosynthesis and may also play a role in disease pathophysiology. Fatty acids themselves may act directly on cells and tissues, causing autophagy and cell death, altering cellular resting membrane potentials and membrane permeability [40]. These cord blood studies have established changes in fetal life at the time of birth, and NBS studies may help determine if these changes persist by virtue of its collection time (in Australia, at least 24–48 h after birth).

A positive association between certain acylcarnitines (AC C4OH) and maternal diabetes phenotypes such as FPG, 1-h glucose, SSF and BW was frequent in both HAPO studies. This finding is relevant to the field of NBS; biochemical variants of AC C4OH and AC C5OH in recent times are more frequently observed in NBS analysis and have been overrepresented over the years [41]. The frequency of these acylcarnitines (AC C4OH and AC C5OH) may be associated with the rising incidence and prevalence of diabetes in pregnancy. Determining the association of certain NBS analytes with intrauterine exposure would be extremely informative from a laboratory perspective.

## 7. Considerations in Using Newborn Screening Results for Diabetes Research

Other groups have explored uses for NBS, such as gestational age dating, risk prediction in neonatal disorders (necrotizing enterocolitis; NEC and sepsis) and survival in prematurity [10,42,43,44,45,46,47,48]. One article hailed NBS as a “hidden treasure” in response to a study that postulated a link between abnormal fatty acid oxidation and mucosal damage in NEC [48]. The potential of NBS in understanding neonatal metabolism and how it links to disease pathology was clearly demonstrated. However, certain caveats exist. The extent of diabetes research using NBS results will depend on the target disorders screened for and the method used. Countries that perform expanded testing using MSMS will be able to perform more in-depth secondary analyses than countries that screen for fewer disorders. The timing of sample collection (day three vs. day five) is crucial, as catabolism slows with advancing age. This is not to say that samples taken on day five would be unsuitable but that it will yield different information about neonatal metabolism and must be taken into account. Consent regarding the secondary use of results/DBS will also vary according to country and may prove to be a limiting factor. For some countries, such as Germany, the “right to not know” is enshrined in law and prohibits the passing information of carrier status of the infant to the parents [49]. This must be carefully considered when diabetes research is undertaken, as certain analytes common to IEM and diabetes may be deranged in asymptomatic carriers (Table 2). 

## 8. Beyond Inborn Errors of Metabolism: Other Biomarkers in Newborn Screening

Technological advances and a better understanding of disease pathophysiology has led to the addition of nonmetabolic disorders to newborn screening. T-cell receptor excision circles (TRECs) and kappa-deleting recombination excision circles (KRECs), by-products of T-cell and B-cell receptor rearrangements, are used to detect primary immune deficiency syndromes and are currently being piloted by the NSW Newborn Screening Programme. Other biomarkers currently in use in newborn screening in other parts of the world include hemoglobin H for thalassemia and sickle cell disease. Precision medicine is rapidly becoming a reality, with risk prediction for certain disorders in the near future potentially modifying diseases. Biomarkers, obtained from other biological samples such as urine, have shown promise in risk stratification, the creation of risk scores combining different measures and disease modeling for type 1 diabetes, thyroid disorders in pregnancy and the effects of GDM on the developing urinary tract [50,51,52]. Perhaps, in the future, NBS results may play a role in disease models as a component of scoring systems involving different clinical and neonatal parameters. 

## 9. Conclusions

The primary purpose of NBS is, and should remain, as screening for conditions that present and require treatment early in life with serious consequences and reasonable frequency. It can be argued that screening for adult-onset disorders in an infant raises anxieties in families, and the benefit of early treatment should be balanced with raised anxieties over additional knowledge. However, there is a wealth of knowledge on physiology that can be acquired and shared across disciplines. The timing of sample collection, which is unique to NBS, provides information on neonatal metabolism with minimal maternal and placental influence during the first few days of life, when the neonate is in a catabolic state. Demystifying metabolism as simultaneous networks and working with biochemical geneticists will aid in the understanding of both common and uncommon disorders. Perhaps the best illustration of these methods in practice is the success of Robert Guthrie himself, whose tireless efforts in the face of significant opposition (his original 1961 paper describing the bacterial inhibition assay was rejected and was not published until 1963) [53] has produced a body of knowledge from which we all continue to profit. Encouraging collaborative research between subspecialties and laboratory sciences would be a good starting point, keeping in mind the dangers of early prediction for adult-onset disorders. Nevertheless, this resource has high potential for the better understanding of the molecular pathways and mechanisms underlying future health and disease. 

## Figures and Tables

**Table 1:** Studies evaluating the metabolic signature of diabetes in newborns in cord blood.

Study/Year	Cohort/Country	Analysis Method	Metabolite Deranged
Type 1 Diabetes			
Oresic et al., 2008 [5]	DIPP/Finland	UPLC/MS GCxGC-TOF/MS	Phosphatidylcholine (↓) Succinic/citric acid (↓)
Oresic et al., 2013 [6]	DiPiS/Sweden	UPLC/MS	Phosphatidylcholine (↓) Succinic/citric acid (↓)
La Torre et al., 2013 [3]	DiPiS	UPLC/MS	Phosphatidylcholine (↓) Phosphatidylethanolamine (↓) Triglycerides (↓)
**Diabetes in Pregnancy**			
Cetin et al., 2005 [9]	Infants of GDM mothers/Milano	HPLC	Valine, Methionine, Phenylalanine, Isoleucine, Leucine, Ornithine, Glutamate (↑) Proline, Alanine (↑) Glutamine (↓)
Dani et al., 2014 [8]	Infants of GDM mothers/Florence	NMRS	Pyruvate, Histidine, Alanine, Valine, Methionine, Arginine, Lysine, α-ketoisovaleric acid, Hypoxanthine, Lipoprotein, Lipid (↑) Glucose (↓)
Fotakis et al., 2016 [10] *	Infants of GDM mothers/Athens	NMRS	Large for gestational age GDM vs. appropriate for gestational age: Valine, leucine, isoleucine, lysine, aCH2, N-acetylglutamine, acetoacetic acid, glutamine/glutamic acid, threonine, creatine and histidine (↑) Large for gestational age GDM vs. large for gestational age non-GDM: glucose, glutamine, valine, histidine, alanine (↑)
Lowe et al., 2017 [11]	HAPO/Mexican-Am, Thai, N Europe, Afri-Carib	MS/MS	Maternal BMI: positive association with BCAA and byproducts, Phenylalanine, AC C3, C4, C5 Maternal Fasting Glucose: C4OH (positive association; Mex-Am only) Maternal 1-h Glucose: positive association with 3OH butyrate/AC-C4OH, Glycerol, AC C10-OH/C8DC Maternal Insulin Resistance: positive association with BCAA and derivatives, AC C3, AC C4/*Ci4,* AC C5, AC C5-DC, AC C4-OH, AC C2, Glycerol, Asparagine/asp (Afri-Car) Subgroup analysis-cord blood c-peptide: negative association with leucine/isoleucine and positive association with AC C5 Newborn Outcomes Birth weight: positive association with Serine, Proline, Glutamine/Glutamate, Glycine, 3OH and AC-C3, C12- OH/C10-OH, C10-OH/C8-DC, C8:1-DC, C6-DC/C8-OH, C8:1-OH/C6:1-DC Negative association with Triglycerides, AC-C20-OH/C18-DC (Thai) Adjusting for cord c-peptide: (in addition to) positive association with Leucine/Isoleucine, Arginine, Ornithine, Citrulline Negative association with AC C4/C*i*4, AC-C20-OH/C18-DC (Thai) AC C8:1, AC C10:3 (Afro-Cari)
Perng et al., 2017 [12]	Project Viva/Massachusetts	UPLC/MS	No association with BCAA or metabolites of energy production and cell proliferation pathways
Patel et al., 2018 [13]	UPBEAT/UK	LC-MS/MS	Adiponectin (↓), Isocitric acid and Lysophosphatidylcholine 18:1 (↑)
Roverso et al., 2019 [14]	Infants of GDM mothers/Padua	ICP-MS	Ca, Cu, Na, Zn (↑), Fe, K, Mn, P, Rb, S, and Si (↓)
**Pharmacotherapy during pregnancy:**			
None looking at acylcarnitines or amino acids on cord blood			

BCAA = Branched chain amino acid, DIPP = type 1 Diabetes Prediction and Prevention Study, DiPiS = Diabetes Prediktion I Skåne, GDM = Gestational Diabetes Mellitus, HAPO = Hyperglycemia and Adverse Pregnancy Outcomes, Mexican-Am = Mexican-American, N Europe = Northern Europe, Afri-Carib = African-Caribbean, UPBEAT = UK Pregnancies and Better Eating Trial, UPLC/MS = Ultra-Performance Liquid Chromatography Mass Spectrometry, GCxGC-TOF/MS = Gas Chromatography Gas Chromatography Time-of-Flight Mass Spectrometry, HPLC = High-Performance Liquid Chromatography, NMR = Nuclear Magnetic Resonance, MS/MS = Tandem Mass Spectrometry (Mass Spectrometry/Mass Spectrometry), LC-MS/MS = Liquid Chromatography Mass Spectrometry and ICP-MS = Inductively Coupled Plasma Mass Spectrometry. * This study compared both maternal and fetal umbilical cord blood and plotting against gestational age and size. Only the umbilical cord blood results of GDM infants are recorded here.

**Table 2:** Selected studies with examples of analyte deranged and the corresponding disorder.

Study	Analyte Deranged	Disorder Tested on NBS
Lowe et al. (2017) [14]	Maternal BMI: Phenylalanine (+ association) AC C3, AC C5, AC C4; Leucine/Isoleucine Cord C-peptide and BW (+association) Arginine Cord C-peptide and SSF AC C4-OH	PKU/Pterin defects Propionic aciduria/methylmalonic aciduria 2-methylbutyrylCoA-dehydrogenase deficiency Isobutyryl CoA-dehydrogenase deficiency Short chain dehydrogenase deficiency Multiple acyl CoA dehydrogenase deficiency Maple syrup urine disease Arginase deficiency Short chain hydroxy acyl CoA dehydrogenase deficiency
Kadakia et al. (2018) [15]	Cord C-peptide (−association) Tyrosine BW (+association) AC C10:1 GDM C16	Tyrosinemia Secondary marker for Medium Chain CoA deficiency Very long chain acyl CoA dehydrogenase deficiency

NBS = newborn screening, BMI = body mass index, AC = acylcarnitine, C3 = proprionylcarnitine, C4 = butyrylcarnitine, C5 = tiglylcarnitine, BW = birth weight, SSF = sum of skinfold thickness, C4-OH = 3-hydroxybutyrylcarnitine, PKU = phenylketonuria, C10:1 = decenoylcarnitine, GDM = gestational diabetes mellitus and C16 = palmitoylcarnitine.

**Table 3:** Characteristics of the selected studies.

Author/Year Study Design	Study Objective	Population Characteristics	Sample Size	Method of Analysis	Results	Comments
Type 1 Diabetes risk and NBS results only						
La Marca, 2013 [7] Case control	To investigate the relationship between carnitines and amino acids with T1DM	50 children from Tuscany and Umbria with T1DM diagnosed ≤5 years; HLA genotyped; Antibody status checked	250 neonates’ NBS results Controls: 200 (same analytic batch)	LC-MS/MS	Lower C2, C3, C4, C5, C14, C16, C18, Total and free carnitine Alanine (*p* ≤ 0.05)	Reported as mean: Total carnitine, acylcarnitine, C2, C5, alanine; Ile and Leu reported as one analyte; CV: none reported
**Reanalyzing for metabolites and T1DM risk**						
Cadario, 2015 [8] Case control	To investigate variations in Vitamin D concentrations at birth and the risk of developing T1DM up to 10 years; potential modifier effect of ethnic groups on the association	Piedmont Diabetes Childhood Registry; 67 children with T1DM 0–10 years	300 neonates’ NBS card 267 controls, matched for birthday (±30 days), place of birth and ethnic group	LC-MS/MS	No association as a whole; 36 cases and 103 controls <2.14; OR 1.76 (0.92–3.38) 31 cases and 133 controls ≥2.14 OR 1.00).	Subgroup analysis: Migrants: 20 cases 31 controls <2.14; OR 14.02 (1.76–111.7); 3 cases 26 controls >2.14; OR 1.0; CV: not reported
Jacobsen, 2016 [9] Case-cohort 2 models (with/out HLA matching)	To investigate low levels of 25(OH)D at birth and the risk of developing type 1 diabetes before the age of 18 years	Danish Childhood Diabetes Registry (DanDiabKids) Case control: 912 Case cohort: 2866	Case-cohort: 3778; Method of choosing controls unspecified; Case control: Model 1–527 pairs Model 2–429 pairs (858 total); These pairs were HLA matched; Controls chosen via DBS card next to index case card	LC-MS	No associations Case cohort: Sub-cohort: (median) 23.8 (15.5, 36.7) cases: 24.3 (14.8, 38.8) Case control: Cases: 21.3 (12.5–33.1) Controls: 21.1 (12.0–32.9)	Both groups used the same registry; overlap with sampling of 4 individuals with T1DM CV: 15%
Kyvsgaard, 2016 [10] Population-based case-control	To investigate association between low perinatal zinc status and the risk of T1DM before 16 years	Danish Childhood Diabetes Register (DanDiabKids)199 cases with T1DM	398 NBS cards 199 controls; Matched by birth year and month	LA-ICP-MS	No association Reference range: 10–19 μmol/L OR: High zinc: 1; Med high zinc: 0.88 (0.41, 1.85) Low zinc: 0.89 (0.40, 1.97)	All samples, negative controls and reference samples analyzed in the same run. Covariates included: Sex, birth year, season, HLADQ1B status, gestational age, birth weight, maternal age at delivery; CV: 15.9%
Kyvsgaard, 2017 [11] Case-control	To investigate association between neonatal iron content and the risk of T1DM before 16 years.	Danish Childhood Diabetes Register (DanDiabKids)199 cases with T1DM HLA-DQB1 genotyping	398 NBS cards 199 controls chosen by consecutive NBS numbers	LA-ICP-MS	Two-fold risk of T1DM with doubling of iron content; Cases (199): 1.80 (0.30); Controls: (199): 1.74 (0.39) OR 2.07 (95% CI) (1.07; 4.00)	All samples analyzed on the same run; After adjusting for confounders (OR 2.55; 1.04; 6.24) CV: 19.3%
**Metabolic signature of monogenic diabetes on DBS**						
McDonald, 2017 [1] Case-control	To assess stability of DBS glucose and the diagnostic accuracy of DBS glucose for neonatal diabetes detection	Newborns part of the Exeter Family Study of Childhood Health; Exeter 10,000 project; infants with genetically confirmed neonatal diabetes (11 cases)	687 infants; 20 volunteers; 170 infants with genetically confirmed neonatal diabetes	UV Spectrometry (manual rate-reaction hexokinase method)	glucose stable in room temp, 4 °C and −20 °C for up to 5 days, stable >14 days in 4 °C and −20 °C; Mean (SD) glucose at day 5 of life: Infants with neonatal diabetes:10.2–>30.0 mmol/L (normal 4.6 mmol (0.7))	CV: 10.3% (3 mmol/L); 15% (14 mmol/L); NBS performed day 5 of life; 5/11 infants with neonatal diabetes diagnosed before NBS performed
Simaite, 2014 [12] Familial linkage, molecular analysis and animal studies	To identify novel diabetes genes	Consanguineous family with nonautoimmune diabetes BIODEF database	Index family 6 families identified through BIODEF	Not specified (usually MS/MS)	Patients with *PCBD1* homozygous mutations may have mild transient hyperphenylalaninemia (>120 umol but <360 umol/L)	2 patients with normal pH levels; Not all patients with homozygous mutations with diabetes; CV not reported
**Metabolic signature of** **diabetes on NBS**						
Sanchez-Pintos, 2017 [13] Observational	To characterize postnatal plasma acylcarnitine profiles in a cohort of LGA newborns. To compare acylcarnitine fingerprint of LGA-GDM vs. LGA-NGDM	All infants born in Hospital	Total N = 2514; SGA: 250; AGA: 2018; LGA: 246 Newborns with GDM exposure: 246 (200 on diet, 46 on insulin); LGA-GDM: 42; LGA-NGDM: 204	MS/MS (derivatized)	For GDM-LGA, Median higher levels of FC, TC, short-chain acylcarnitines incl C3, lower levels of medium and long-chain acylcarnitines-NS	No information on amino acids; No information on degree of diabetes control during pregnancy

TIDM = Type 1 diabetes mellitus, HLA = human leukocyte antigen, NBS = newborn screening, LC = liquid chromatography, MS = mass spectrometry, C2 = acylcarnitine, C3 = propionylcarnitine, C4 = butyrylcarnitine, C5 = isovalerylcarnitine, C14 = tetradecanoylcarnitine, C16 = palmitoylcarnitine, C18 = stearoylcarnitine, CV = coefficient of variation, OR = odds ratio, 25 (OH)D = 25 hydroxy vitamin D, LA-ICP-MS = laser ablation inductively coupled plasma mass spectrometry, UV = ultraviolet, SD = standard deviation, *PCBD1* = pterin-4 alpha-carbinolamine dehydratase 1, LGA = large for gestational age, GDM = gestational diabetes mellitus, SGA = small for gestational age, AGA = appropriate for gestational age and NS = nonsignificant.

**Table 4:** Comparison of NBS DBS (whole blood) vs. cord blood as a sample.

Sample Characteristics	NBS DBS	Cord Blood	Comments
**Components**	whole blood	plasma	DBS samples need correcting for hematocrit
**Volume**	50–75 μL per blood spot	60–110 mL	Small volume of DBS in NBS limits use in untargeted metabolomic techniques
**Storage**	Once dry, may be stored at room temperature	Needs special storage facilities to keep temperature between −70 to −20 °C.	DBS more prone to measurements of uncertainty such as transport conditions, weather, etc.
**Timing of collection**	At least 24 h following delivery with some countries testing between day 3–5 following delivery	Collected immediately after delivery	Cord blood samples may reflect placental and maternal metabolism while the neonate is receiving a constant supply of nutrition. NBS DBS reflect infants’ metabolism (independent of maternal and placental influence) with periods of fasting in between feeding.
**Coverage**	Near universal in countries that have NBS programs (>99% in NSW, Australia) incorporated into public health	Variable; usually collected as part of a research project or private cord blood banking	Countries with well-established NBS programs within a framework of socialized medicine are able to draw on NBS DBS for uses outside research (i.e., source of DNA for retrospective cascade testing), forensic medicine, and quality assurance programs
**Metabolic intermediates tested**	Acylcarnitines, free and total acylcarnitines, some amino acids, 17 hydroxyprogesterone, thyrotropin, trypsinogen, galactose Conditions tested (and metabolites) vary among programs	Wide spectrum of intermediates may be tested, including phospholipids and their intermediaries, ceramides, intermediaries of various metabolic networks	NBS testing from DBS in NSW includes testing for other conditions (muscular dystrophies, immune deficiency syndromes, cystic fibrosis). Feasibility of antibody testing on DBS for type 1 diabetes has been established. Volume and storage conditions of cord blood allow discovery and identification of metabolic intermediates not previously described.

DBS = dried blood spot, DNA = deoxyribonucleic acid, NBS = newborn screening, and NSW = New South Wales.

## Supplementary Materials

The following are available online at https://www.mdpi.com/2073-4409/9/10/2299/s1.
