# Supplementary material for: Newborn Screening Samples for Diabetes Research: An Underused Resource
Source: Cells. 2020 Oct 15;9(10):2299. doi: 10.3390/cells9102299 (PMC7602529; doi:10.3390/cells9102299)
Supplement: Supplementary file 1 [file cells-09-02299-s001.pdf]

Supplementary Table S1. Search strategy for research objective 1

Research objective 1: To explore the association between newborn screening analytes and the development of T1DM

	Search	Results
**Cochrane Library search: 29/3/19**		
With Cochrane Library publication date from Apr 1998 to Dec 2018		
#1	type 1 diabetes OR diabetes OR T1DM	54925
#2	Mass spectrometry OR tandem spectrometry OR LC-MS OR GC-MS OR Nuclear Magnetic Resonance Spectrometry OR metabolomics OR metabolic profile OR metabolites OR carnitines OR acylcarnitines OR fatty acid oxidation	13220
#3	#1 AND #2	1837
#4	Newborn screening OR dried blood spots OR heelprick OR Guthrie cards	1361
#5	#1 AND #2 AND #4	20
**Ovid Medline search: 29/3/19**		
1	type 1 diabetes.ab.	48684
2	diabetes.ab.	650846
3	t1DM.ab.	7894
4	1 or 2 or 3	651527
5	Newborn screening.ab.	7124
6	dried blood spots.ab.	3139
7	heelprick.ab.	17
8	Guthrie cards.ab.	286
9	5 or 6 or 7 or 8	9835
10	Mass spectrometry.ab.	229478
11	tandem spectrometry.ab.	51
12	LC-MS.ab.	52523
13	GC-MS.ab.	43000
14	Nuclear Magnetic Resonance Spectrometry.ab.	322
15	metabolomics.ab.	15972
16	metabolites.ab.	202388
17	metabolic profile.ab.	8272
18	carnitines.ab.	569
19	acylcarnitines.ab.	2184
20	fatty acid oxidation.ab.	10051
21	10 or 11 or 12 or 13 or 14 or 15 or 16 or 17 or 18 or 19 or 20	479902
22	4 and 9 and 21	16
**PubMed search: 29/3/19**		
#1	((type 1 diabetes) OR diabetes) OR TIDM	662858
#2	newborn screening OR dried blood spots OR heelprick OR Guthrie cards	35679
#3	mass spectrometry OR tandem mass spectrometry OR LC-MS OR GC-MS OR NMRS OR Nuclear Magnetic Resonance Spectrometry OR metabolomics OR metabolic profile OR metabolites OR carnitines OR acylcarnitines OR fatty acid oxidation	723913
#4	(((((type 1 diabetes) OR diabetes) OR T1DM)) AND ((mass spectrometry OR tandem mass spectrometry OR LC-MS OR GC-MS OR NMRS OR Nuclear Magnetic Resonance Spectrometry OR metabolomics OR metabolic profile OR metabolites OR carnitines OR acylcarnitines OR fatty acid oxidation))) AND ((newborn screening OR dried blood spots OR heelprick OR Guthrie cards))	47

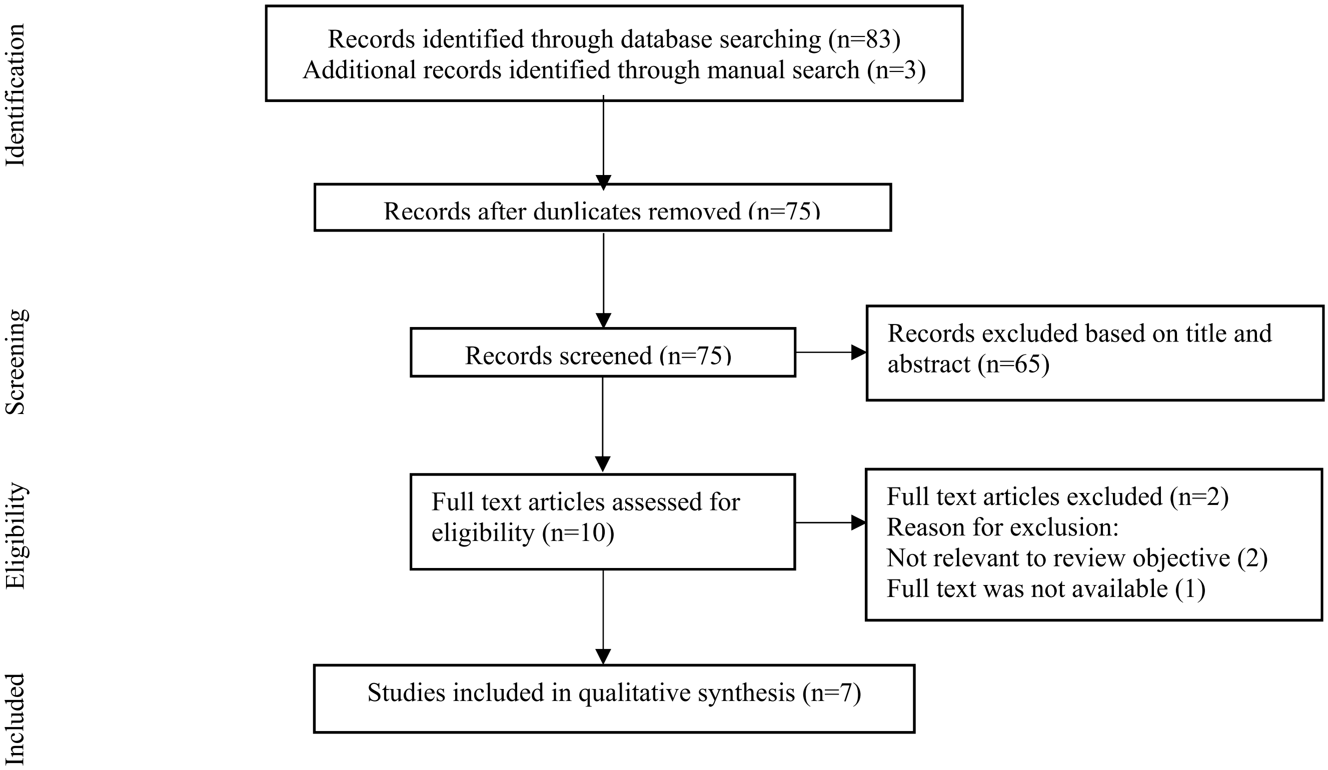

Figure S1. Flow chart of selected studies for objective one.

Supplementary Table S2. Search strategy for research objective 2

Research objective 2: To determine the association between newborn screening analytes and

i) exposure to diabetes in pregnancy;

ii) treatment of diabetes in pregnancy.

	Search	Results
**Cochrane Library search: 29/3/19**		
With Cochrane Library publication date from Apr 1998 to Dec 2018		
#1	diabetes in pregnancy OR pregnancy in diabetes OR diabetes OR gestational diabetes OR type 1 diabetes OR type 2 diabetes OR pregnancy OR ODIP OR Overt diabetes in pregnancy<br>with Cochrane Library publication date from Apr 1998 to Dec 2018	83815
#2	offspring OR infants OR Newborn screening OR dried blood spots OR heelprick OR Guthrie cards	27734
#3	mass spectrometry OR tandem mass spectrometry OR LC-MS OR GC-MS OR NMRS OR Nuclear Magnetic Resonance Spectrometry OR metabolomics OR metabolic profile OR metabolites OR amino acids OR branched-chain amino acids OR carnitines OR acylcarnitines OR fatty acid oxidation	18755
#4	#1 AND #2 AND #3	190
**EMBSAE search: 30/3/19**		
1	type 1 diabetes	48684
2	diabetes.ab.	650846
3	t1DM.ab.	7894
4	Newborn screening.ab.	7124
5	dried blood spots.ab.	3139
6	heelprick.ab.	17
7	Guthrie cards.ab.	286
8	Mass spectrometry.ab.	229478
9	tandem spectrometry.ab.	51
10	LC-MS.ab.	52523
11	GC-MS.ab.	43000
12	Nuclear Magnetic Resonance Spectrometry.ab.	322
13	metabolomics.ab.	15972
14	metabolites.ab.	202388
15	metabolic profile.ab.	8272
16	carnitines.ab.	569
17	acylcarnitines.ab.	2184
18	fatty acid oxidation.ab.	10051
19	diabetes in pregnancy.ab.	1097
20	pregnancy in diabetes.ab.	29
21	gestational diabetes.ab.	18510
22	type 2 diabetes.ab.	151181
23	pregnancy.ab.	422162
24	ODIP.ab.	4
25	Overt diabetes in pregnancy.ab.	19
26	1 or 2 or 19 or 20 or 21 or 22 or 23 or 24 or 25	1047454
27	offspring.ab.	77614
28	infants.ab.	280881
29	4 or 5 or 6 or 7 or 27 or 28	362604
30	NMRS.ab.	311
31	8 or 9 or 10 or 11 or 12 or 13 or 14 or 15 or 16 or 17 or 18 or 30	480154
32	amino acids.ab.	212427
33	branched-chain amino acids.ab.	4515
34	31 or 32 or 33	677310
35	26 and 29 and 34	1154
**PubMed search : 30/3/19**		
#1	(pregnancy in diabetes[Title/Abstract] OR diabetes in pregnancy[Title/Abstract] OR diabetes[Title/Abstract] OR gestational diabetes[Title/Abstract] OR type 1 diabetes[Title/Abstract] OR type 2 diabetes[Title/Abstract] OR pregnancy[Title/Abstract] OR ODIP[Title/Abstract] OR Overt diabetes in	439599

	pregnancy[Title/Abstract]) Filters: Publication date from 1998/04/01 to 2018/12/31; Humans	
#2	(offspring[Title/Abstract] OR infants[Title/Abstract] OR Newborn screening[Title/Abstract] OR dried blood spots[Title/Abstract] OR heelprick[Title/Abstract] OR Guthrie cards[Title/Abstract]) Filters: Publication date from 1998/04/01 to 2018/12/31; Humans	144388
#3	(mass spectrometry[Title/Abstract] OR tandem mass spectrometry[Title/Abstract] OR LC-MS[Title/Abstract] OR GC-MS[Title/Abstract] OR NMRS[Title/Abstract] OR Nuclear Magnetic Resonance Spectrometry[Title/Abstract] OR metabolomics[Title/Abstract] OR metabolic profile[Title/Abstract] OR metabolites[Title/Abstract] OR amino acids[Title/Abstract] OR branched-chain amino acids[Title/Abstract] OR carnitines[Title/Abstract] OR acylcarnitines[Title/Abstract] OR fatty acid oxidation[Title/Abstract]) Filters: Publication date from 1998/04/01 to 2018/12/31; Humans	162630
#4	(((((pregnancy in diabetes[Title/Abstract] OR diabetes in pregnancy[Title/Abstract] OR diabetes[Title/Abstract] OR gestational diabetes[Title/Abstract] OR type 1 diabetes[Title/Abstract] OR type 2 diabetes[Title/Abstract] OR pregnancy[Title/Abstract] OR ODIP[Title/Abstract] OR Overt diabetes in pregnancy[Title/Abstract])) AND ("1998/04/01"[PDat] : "2018/12/31"[PDat]) AND Humans[Mesh])) AND (((offspring[Title/Abstract] OR infants[Title/Abstract] OR Newborn screening[Title/Abstract] OR dried blood spots[Title/Abstract] OR heelprick[Title/Abstract] OR Guthrie cards[Title/Abstract])) AND ("1998/04/01"[PDat] : "2018/12/31"[PDat]) AND Humans[Mesh])) AND (((mass spectrometry[Title/Abstract] OR tandem mass spectrometry[Title/Abstract] OR LC-MS[Title/Abstract] OR GC-MS[Title/Abstract] OR NMRS[Title/Abstract] OR Nuclear Magnetic Resonance Spectrometry[Title/Abstract] OR metabolomics[Title/Abstract] OR metabolic profile[Title/Abstract] OR metabolites[Title/Abstract] OR amino acids[Title/Abstract] OR branched-chain amino acids[Title/Abstract] OR carnitines[Title/Abstract] OR acylcarnitines[Title/Abstract] OR fatty acid oxidation[Title/Abstract])) AND ("1998/04/01"[PDat] : "2018/12/31"[PDat]) AND Humans[Mesh]) Filters: Publication date from 1998/04/01 to 2018/12/31; Humans	443

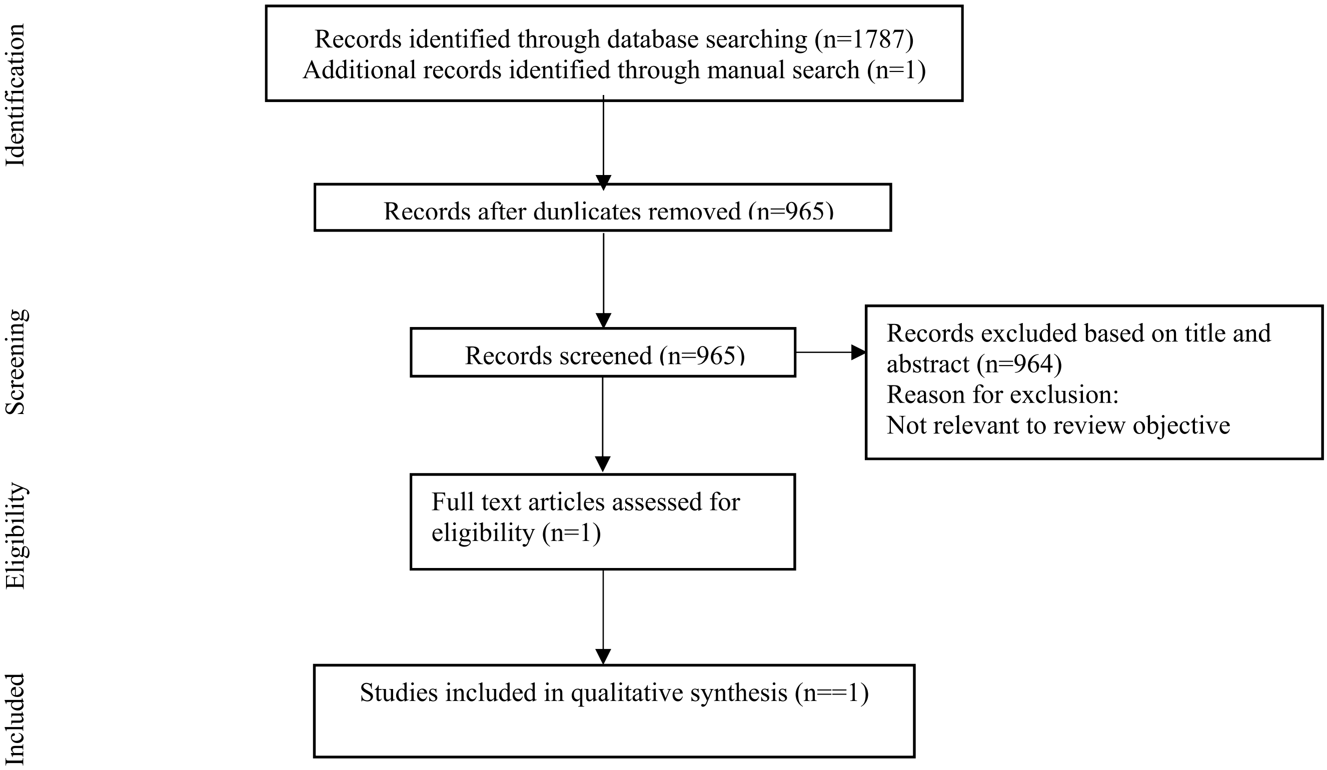

Figure S2. Flow chart of selected studies for objective two.
